# Supporting and strengthening research on urban health interventions for the prevention and control of vector-borne and other infectious diseases of poverty: scoping reviews and research gap analysis

**DOI:** 10.1186/s40249-018-0462-z

**Published:** 2018-09-03

**Authors:** Mariam Otmani del Barrio, Frédéric Simard, Andrea Caprara

**Affiliations:** 1 Vectors, Environment and Society Unit, Special Programme for Research and Training in Tropical Diseases (TDR), World Health Organization (WHO), 20, avenue Appia, CH-1211 Geneva 27, Switzerland; 2 Maladies Infectieuses et Vecteurs: Ecologie, Génétique, Evolution et Controle (MIVEGEC), IRD-CNRS University of Montpellier, Montpellier, France; 3 Ceará State 639 University, Fortaleza, Brazil

**Keywords:** Vector-borne diseases, Urban health interventions, Climate change, Effectiveness, Research priority setting, Surveillance, Vector control

## Abstract

**Background:**

More than half of the world’s population currently lives in urban settlements that grow both in size and number. By 2050, approximately 70% of the global population will be living in urban conglomerations, mainly in low- and middle-income countries. Mobility, poverty, different layers of inequalities as well as climate variability and change are some of the social and environmental factors that influence the exposure of human populations in urban settings to vector-borne diseases, which pose eminent public health threats. Accurate, consistent, and evidence-based interventions for prevention and control of vector-borne and other infectious diseases of poverty in urban settings are needed to implement innovative and cost-effective public policy and to promote inclusive and equitable urban health services.

**Main body:**

While there is growing awareness of vector-borne diseases epidemiology at the urban level, there is still a paucity of research and action being undertaken in this area, hindering evidence-based public health policy decisions and practice and strategies for active community engagement. This paper describes the collaboration and partnership of the Special Programme for Research and Training in Tropical Diseases (TDR) hosted by the World Health Organization (WHO) and the “VEctor boRne DiseAses Scoping reviews” (VERDAS) Research Consortium as they joined efforts in response to filling this gap in knowledge and evidence by supporting the development of a series of scoping reviews that highlight priority research gaps and policy implications to address vector-borne and other infectious diseases at the urban level.

**Conclusions:**

The set of scoping reviews proposed in this special issue presents a critical analysis of the state-of-the-art of research on urban health interventions for the prevention and control of vector-borne and other infectious diseases of poverty. The authors of the 6 reviews highlighted severe gaps in knowledge and identified organizational and theoretical limitations that need to be urgently tackled to improve cities preparedness and vector control response. The more pressing need at present is to ensure that more implementation research on vector-borne diseases in urban settings is conducted, addressing policy and practice implications and calling for more political commitment and social mobilization through adequate citizen engagement strategies.

**Electronic supplementary material:**

The online version of this article (10.1186/s40249-018-0462-z) contains supplementary material, which is available to authorized users.

## Multilingual abstracts

Please see Additional file 1 for translations of the abstract into the six official working languages of the United Nations.

## Background

Vector-borne diseases account for around 17% of all infectious diseases, with the highest burden of these diseases in tropical and subtropical regions, which affect disproportionately less resourced populations [1]. Lyme disease and other tick-borne diseases are however spreading speedily in temperate regions of the globe and the worldwide incursion into urban areas of *Aedes* mosquitoes presents new threats for the emergence and burst of arboviruses. Today, more than half of the world’s population lives in urban settlements that grow both in size and number. By 2050, approximately 70% of the global population will be living in urban conglomerations, mainly in low- and middle-income countries (LMICs) [2].

Global demographic mobility and trade, poverty, different layers of inequalities as well as climate variability and change are some of the social and environmental factors that influence the exposure of human populations in urban settings to vector-borne diseases (VBDs) such as dengue, chikungunya and Zika virus diseases, urban malaria, leishmaniasis, and lymphatic filariasis, apart from water-borne diseases among others. These VBDs pose eminent public health challenges with emerging and re-emerging infections, particularly in the era of globalization and interconnectedness, requiring strengthened intersectoral policies, interventions and commitments at the urban level.

Accurate, consistent, and evidence-based interventions for prevention and control of VBDs and other infectious diseases of poverty in urban settings are needed to implement cost-effective public policy and to promote inclusive, equitable and sustainable urban health services.

Urban health is influenced by several factors including governance, population features, urban planning, and socioeconomic development and health services, among others, which in turn have major implications for social and environmental determinants of health. With the growing rate of urbanization, major public health challenges remain and are likely to be exacerbated, ranging from infectious diseases such as VBDs and water-borne diseases to non-communicable diseases (e.g. respiratory diseases) [3, 4] that continuously threaten human health and equity targets. The existence of small and medium towns, and the growth of urban slums, including highly ignored *non-notified slums*, often lacking reliable and safe piped water, adequate solid waste management and other basic services, can render large populations in towns and cities at risk of VBDs such as mosquito-borne diseases [5, 6]. The risk of infection is particularly high in towns and cities where vectors proliferate and where contact with human beings is high. The disease burden is often disproportionately high in poorer communities, where malnourished populations with weakened immunity are especially susceptible. Altogether, VBDs cause more than 1 million deaths each year [5].

The incidence and distribution of VBDs is consequently influenced by social, demographic and environmental factors that interact under a changing climate and affect pathogen transmission patterns. This results in an intensification, geographical spread, re-emergence or extension of transmission seasons [7] from which cities, especially in resource constraint settings with poor health-promoting policies, are not immune.

Integrated and comprehensive approaches are required to prevent, detect, report and respond to outbreaks of VBDs globally, as highlighted in the recent Resolution WHA70.16 on the Global Vector Control Response 2017–2030 adopted by World Health Organization (WHO) Member States in June 2017 at the World Health Assembly, which calls countries to develop or adapt existing vector control strategies and operational plans at national level to align them to this integrated strategic approach. In line with growing evidence that demands more attention and innovation on mobilizing participation of the urban communities for health improvement along with transdisciplinary collaboration [8], this resolution requests countries to ensure active community engagement and more research and innovation. The Resolution also serves as a supporting mechanism to strengthen technical capacity, monitoring and surveillance and enhance infrastructure. Whereas this is a renovated effort to strengthen prevention and control of VBDs at the global level, it also highlights the importance of multisectoral collaboration beyond the health sector. Other sectors, including environment, urban planning and housing and education are key milestones for health improvement at the urban level in general, and in particular they are critical to ensure cost-effective and integrated responses to fight multiple vectors and diseases and their consequent public health challenges [5]. This cooperation is also critical to ensure that vector control is planned and implemented in a timely manner, adequately and sustained in time.

The Special Programme for Research and Training in Tropical Diseases (TDR)^2^ and the “VEctor boRne DiseAses Scoping reviews” (VERDAS) Research Consortium have joined hands to fill gaps in knowledge and evidence by supporting the development of a series of scoping reviews on urban health and VBDs and other infectious diseases of poverty. Knowledge generated from the scoping reviews is expected to contribute to a better understanding of the priority research gaps and policy implications in this area and improve the ability of urban settings to address VBDs and other infectious diseases.

## Main text

In 2015, TDR launched a call inviting research groups or consortia from worldwide institutions to express interest in support of a long-term effort to strengthen research on urban health interventions for the control of vector-borne and other infectious diseases of poverty.

VERDAS research Consortium, for “VEctor boRne DiseAses Scoping reviews”, was established in response to this call issued by the Vectors, Environment and Society Unit of TDR hosted at WHO. The overall objective of the call and research initiative were to conduct a knowledge gap analysis and research prioritization exercise on the basis of a series of six state-of-the-art scoping reviews and subsequently identify implications for policy and practice.

Through this process, TDR brought together global experts convened by the VERDAS Research Consortium to generate evidence on urban health interventions that address social and environmental determinants of health, and to conduct a research gap analysis, including a series of scoping reviews and an expert consultation to identify research priorities regarding urban health interventions for the prevention and control of vector-borne and other infectious diseases of poverty. This special issue draws together the resulting scoping reviews and the ideas presented at the workshop consultation and helps focus attention on the research gaps and policy implications that need to be considered to address VBDs and other infectious diseases at the urban level.

Twenty-seven researchers and one research coordinator from various research institutions from Brazil, Burkina Faso, Canada, Colombia, France and Spain constituted the consortium (Table 1). Each review was distributed among the researchers according to their expertise.

**Table 1:** List of urban health and vector-borne diseases scoping reviews included in this special issue and corresponding research teams

	Scoping Leader	Institution	Research team	Title of the review
1	Lyda Osorio	Universidad del Valle. Cali, Colombia	Lyda Osorio, Jonny Alejandro Garcia, Luis Gabriel Parra, Victor Garcia, Laura Torres, Stéphanie Degroote, Valery Ridde	A scoping review on the field validation and implementation of rapid diagnostic tests for vector-borne and other infectious diseases of poverty in urban areas
2	Florence Fournet	Institut de Recherche pour le Développement. Montpellier, France	Florence Fournet, Frédéric Jourdain, Emmanuel Bonnet, Stéphanie Degroote, Valéry Ridde	Effective surveillance systems for vector-borne diseases in urban settings and translation the data into action: a scoping review
3	Clara Bermudez-Tamayo	Escuela Andaluza de Salud Publica. Grenanda, Spain	Jorge Marcos-Marcos, Antonio Olry de Labry-Lima, Silvia Toro-Cardenas, Marina Lacasaña, Stéphanie Degroote, Valéry Ridde, and Clara Bermudez-Tamayo	Impact, economic evaluation and sustainability of integrated vector management in urban settings to prevent vector-borne diseases: a scoping review
4	Marcus Eder and Celina Maria Turchi Martelli	Fundação Oswaldo Cruz. Recife, Brazil	Marcus Eder, Fanny Cortes, Noêmia Teixeira de Siqueira Filha, Giovanny Vinícius Araújo de França, Stéphanie Degroote, Cynthia Braga, Valéry Ridde, Celina Maria, Turchi Martelli	Scoping review on vector-borne diseases in urban areas: transmission dynamics, vectorial capacity and co-infection
5	Kate Zinszer and Mabel Carabali	Université de Montréal. Montréal, QC, Canada	Laurence Campeau, Stéphanie Degroote, Valery Ridde, Mabel Carabali, Kate Zinszer	Containment measures for emerging and re-emerging vector-borne and other infectious diseases of poverty in urban settings: a scoping review
6	Stéphanie Degroote	Université de Montréal. Montréal, QC, Canada	Stéphanie Degroote, Kate Zinzser, Valery Ridde	Interventions for vector-borne diseases focused on housing and hygiene in urban areas: a scoping review

**Fig. 1:**
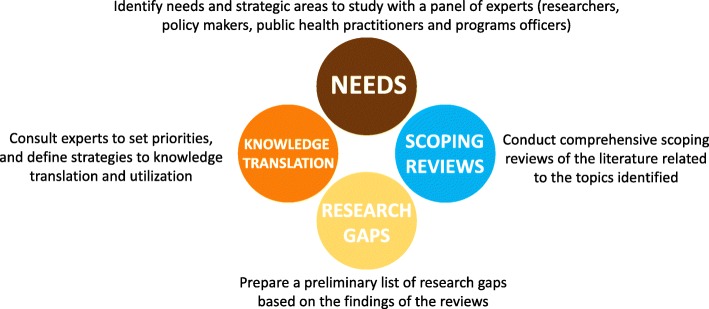
Process followed from identifying research needs to knowledge translation. Courtesy of VERDAS Research Consortium

The research gap and prioritization activities conducted by the different teams followed three phases (Fig. 1): (i) e-Delphi exercise; (ii) protocol development; (iii) multi-stakeholder expert consultation workshop. First, the project started with an e-Delphi exercise with a 3-round consultation to identify research needs and define the six research themes for each review. This e-Delphi consultation took place involving more than one hundred multidisciplinary experts including researchers, public health policy makers, public health practitioners and programme officers and representatives from the private sector working in vector control strategies.

Once the six top priority topics of research were obtained, these were evaluated by each research team leader and formulated into 6 research questions for each review to be performed by the VERDAS consortium.

Second, a protocol was developed to ensure work harmonization across teams and lastly, a workshop was held at Universidad del Valle, Cali (Colombia) with participants from both scientific and policy-based institutions to enable knowledge exchange and to identify research priorities based on knowledge gaps identified in the scoping reviews, and that emerged following a concept mapping exercise.^1^ The workshop served to: (i) share preliminary results of the six scoping reviews, (ii) perform a concept mapping to list and prioritize knowledge gaps and research needs identified in the six scoping reviews, (iii) initiate the development of information briefs highlighting implications for policy and practice for each review.

The key themes that emerged from the e-Delphi exercise for the scoping reviews cover the following areas (Table 1): Field validation and implementation of rapid diagnostic tests; Effective surveillance systems; Impact, economic evaluation and sustainability of Integrated Vector Management; Transmission dynamics, vector capacity and co-infection; Containment measures of emerging and re-emerging vector-borne and other infectious diseases of poverty; Housing and hygiene interventions to prevent vector-borne diseases. A brief overview of each of these six themes covered by the scoping reviews in this special issue is presented in the subsections below.

### A scoping review on the field validation and implementation of rapid diagnostic tests for vector-borne and other infectious diseases of poverty in urban areas [9]

In acute infectious diseases, prompt diagnosis, particularly within 72 h of fever onset, is critical. Differentiating Zika virus, dengue and chikungunya from other common febrile illnesses is difficult and there is a need for simple and cost-effective laboratory tests to support early and accurate diagnosis. The scoping review of Lyda Osorio et al. explores and summarizes the evidence on field validation and implementation in urban areas of rapid diagnostics for VBDs and other infectious diseases of poverty. The manuscript presents a major review mostly of malaria rapid diagnostic tests. The conclusions of the paper present the key research topic areas for future systematic reviews, new research agendas and actions for new vector control policies: evidence comes from malaria tests while rapid tests for tuberculosis and visceral leishmaniasis require further implementation studies. It is emphasized that more evidence on performance of current tests or development of new alternatives is needed also for dengue, Chagas disease, filariasis, leptospirosis, enteric fever, human African trypanosomiasis, schistosomiasis and cholera.

Results from this study would be useful to health care providers having to choose among several different rapid diagnostics options and may contribute to improve clinical management and diagnosis of VBDs. Performance studies were carried out in several countries of various continents, but local studies of malaria diagnostics may be required, since results from a specific region are context specific. Conclusions from this review clearly highlight the need to take context into consideration when deciding on the use of rapid diagnostics, as performance, impact, and implementation outcomes are highly variable. Authors also emphasize the importance of considering the beliefs of communities and providers before implementing rapid diagnostics and highlight important considerations for public health policy and practice before, during and after implementation.

### Effective surveillance systems for vector-borne diseases in urban settings and translation the data into action: a scoping review [10]

The era of globalization and urbanization is revolutionizing the epidemiology of VBDs worldwide, allowing the emergence of new infectious threats and re-emergence of old deadly foes like malaria and dengue. Vectors and pathogens in human transportations are spreading at an ever-increasing speed all over the planet. In this very dynamic system, cities are the nodes of a network where new encounters between vectors, pathogens and susceptible human populations in high numbers provide opportunities for rapid epidemic outbreaks, sometimes with dramatic public health consequences. Effective surveillance systems are thus required to ensure adequate and well-timed responses to VBDs in urban settings and to prevent further spread of the disease. In this context, the aims of the review by Fournet et al. were to establish the state-of-the-art of VBDs surveillance systems in urban environments and to assess their potential to inform public health policies and transform data into effective control interventions.

Their extensive literature search identified 79 documents meeting their inclusion criteria after full-text screening and quality checking. Arboviral diseases and their vectors were the targets of two thirds of these studies. Malaria was the focus of a dozen of studies from Africa and one from India. The review assessed highly diverse surveillance systems, involving active and/or passive surveillance, targeting either the vector or the pathogen in its human host, although rarely monitoring both in an integrated way. Furthermore, few studies provided information about the target population and/or financial and human resources involved, and this was highlighted as a major roadblock to transferability. Improving study designs using standardized data collection and management tools, and developing robust theoretical grounds for interventional research, were identified as research priorities, together with the need for innovative research to foster the development of new tools for vector and pathogen control, resistances mitigation, and identification of residual sources of infection.

The authors further propose that the use of cost-effective technologies such as Geographic Information System and mobile phones appears promising to reduce the time lag between data collection and their translation into control actions as well as for increasing population awareness and mobilization, which are keys to intervention efficiency and sustainability. Institutional support and partner mobilization were also highlighted as key elements for intervention success and should be facilitated by the formalization and implementation of dedicated cross-sectoral coordination structures. Collaboration within the health sector needs to be streamlined, and innovative intersectoral partnerships (e.g. infrastructure construction, urban planning, or water and sanitation) incorporating private companies need to be developed. High-level advocacy and legislation are encouraged as means for increasing political commitment, favouring engagement by health and urban policy actors, and further reducing time lag between data collection and dissemination. Capacity building through staff training and infrastructure development is a requisite not only at the national but also at the local level, to ensure timely case detection and reporting using appropriate tools and guidelines that need to be adapted to the local context, but based on shared rules and known by every actor in the decision chain.

### Impact, economic evaluation and sustainability of integrated vector management in urban settings to prevent vector-borne diseases: a scoping review [11]

According to the WHO, Integrated Vector Management (IVM) is a rational decision-making process for the optimal use of resources for vector control which aims to improve the efficacy, cost-effectiveness, ecological soundness and sustainability of disease-vector control [12]. The IVM strategy is based on the premise that various public and private agencies, including communities, have to be involved in vector control. Vector control programmes in endemic countries are then encouraged to establish and implement national policies to support IVM. The aim of Marcos-Marcos et al. was to identify components related to impacts, economic evaluation, and sustainability that may facilitate implementation of an IVM approach in urban settings to prevent vector-borne diseases. At the end of the extraction process, 42 documents were reviewed of which 30 focused on dengue vectors, eight on malaria, and two on leishmaniasis. More than a half of the studies were conducted in the Americas.

The scoping review highlights research gaps and the scarcity of countries with operational IVM. Results also underlined the lack of robust studies like randomized controlled trials to allow evaluation of the implementation process of interventions. In the same way, the quasi absence of IVM economic evaluation was stressed. Future research should further embrace both the need for evidence-based studies integrating the local context and the possibility of transferring the results to other contexts, calling for the use of qualitative and mixed methods.

Furthermore, health outcomes should be comprehensively assessed. Though illness incidence is a key factor to determine the cost-effectiveness of an intervention in a specific context, it should not be the only indicator used. For example, Worobey et al. showed that outdoor biting of *Aedes albopictus*, vector of dengue, may contribute to child obesity by reducing physical activity [13]. Such finding highlights the need to consider the social determinants of health which could allow the appraisal of health inequalities. Using this approach might facilitate selection and targeting of vector control interventions.

The technical and operational sustainability of vector control strategies is of major concern, given the threat of insecticide resistance, and given the current dependence on external funding, particularly in malaria control. Ensuring sustainability and conducting economic evaluation in the long run appears to be of paramount relevance. However, achieving sustainability clearly requires taking into account lasting affordability of the IVM in the community and the environment [14]. Community involvement was highlighted as a major key to vector control success, requiring consolidated capacity building for sustainability. Finally, the authors advocate for planners as well as researchers to adopt a more coordinated, multi-disease strategy for vector control in line with recommendations from the Global Vector Control Response 2017–2030 [15].

### Scoping review on vector-borne diseases in urban areas: transmission dynamics, vectorial capacity and co-infection [16]

Urban and periurban settings form a complex and heterogeneous environment. It is therefore essential to precisely characterize the transmission of pathogens in this specific context to adapt surveillance and control of VBDs. This issue was addressed by Eder et al. in a scoping review aiming to draw up the state of current knowledge on transmission dynamics, vector capacity, and co-infections regarding VBDs in urban areas.

Articles that met the inclusion criteria dealt mainly with dengue or malaria. Dengue transmission in urban areas was investigated in Asia and America whereas malaria research was mostly performed in Africa, in line with the global epidemiology of these mosquito-borne diseases.

Knowledge gaps were identified, including the role of asymptomatic individuals, the impact of co-infections, and the importance of environmental factors, such as climate variability and change as well as other socio-economic factors on VBDs transmission. Co-infections are only addressed in two studies, both dealing with malaria. Both studies highlight the general lack of knowledge on this phenomenon in different fields such as immunology, clinic, diagnosis and treatment. However, co-infections are not limited to malaria and should be more systematically considered given the impact on diagnosis strategies, and vaccine development challenges [17].

Concerning dengue, the main topics tackled the relationship between the incidence of dengue cases and vector density and human mobility, the role of asymptomatic virus carriers in the spread of the disease, and the impact of climatic conditions on vector abundance. The introduction of imported viremic cases into non-endemic urban areas was identified as a critical issue in this kind of setting. Urban environments are characterized by marked heterogeneities in transmission patterns occurring not only in space but also in time, often at the finest grain [18]. The detection of epidemic phenomena is further undermined by the high human density, human mobility and the proportion of asymptomatic infections. Moreover, traditional entomological indicators appear of limited value, and therefore intake, for public health management, as contradictory results were retrieved. Other risk factors for dengue transmission appear more clearly. This is the case of living and working conditions, as illustrated by differences in the epidemiology of dengue on both sides of the border between the United States and Mexico [19].

Malaria, on the other hand, has been predominantly considered as a rural disease. However, due to their high population size and short generation time, vectors and pathogens rapidly adapt to new environmental conditions in Africa: colonization of highly polluted urban centers by once-rural, insecticide-resistant anopheline mosquitoes is being reported, announcing shifts in disease transmission and epidemiology. Henceforth, urban malaria transmission clearly represents a major challenge for public health, especially in Africa [20]. In urban settings, vector control strategies can be different from those deployed in rural areas. For example, in certain circumstances, identification and elimination of breeding sites could be favored to long-lasting insecticidal nets and indoor residual spraying in urban areas [21]. Monitoring and evaluation of vector control interventions is critical and might then benefit from increased adherence to social media in urban areas. Social media may have potential to facilitate real-time monitoring of spatiotemporal variations in transmission as well as to assess the population’s knowledge, perceptions and practices through citizen science. However, the emerging use of social media will have to deal with human representation and timely detection of unexpected events [22].

Many recommendations are proposed for public health policies and practices, including targeting the most at-risk populations by routine vector control and using a syndromic approach for multi-diseases surveillance to allow timely detection of emerging pathogens and early outbreaks.

### Containment measures for emerging and re-emerging vector-borne and other infectious diseases of poverty in urban settings: a scoping review [23]

Campeau et al. focus on addressing the capacity of systems to respond to emerging diseases and what knowledge gaps originate from emerging epidemics to contain future outbreaks particularly in cities, with high vector density and urban areas with low-income.

Authors verified the evidence on the effectiveness of containment measures for emerging and re-emerging VBDs and other infectious diseases of poverty in urban settings. They also identified gaps and limitations calling for more research, and highlighted implications for public health practice.

Authors emphasize that the largest body of evidence concerned control interventions for Ebola virus and dengue fever, including multiple types of measures categorized in four groups: i) healthcare provision; ii) epidemiological investigation and/or surveillance; iii) environmental or sanitary interventions; and iv) community-based interventions. The results of this scoping review clearly demonstrate that evidence for the effectiveness of containment interventions is very limited. Campeau et al. highlight that one-third of the studies did not provide a clear description of the outcomes and of the procedures or tools used for the intervention, concluding that studies should extend beyond solely reporting on effectiveness and urge to take into account the complexity of real-world settings. An important consideration arising from this review emphasizes the need for more extensive follow-up and multiple information sources to better understand the possible causality of interventions given the existing challenges for establishing causation when assessing the effect of containment measures.

Reinforcing the training of doctors and other health professionals on the diagnosis, management, and treatment of emerging and re-emerging diseases, increasing available resources for disease containment, and improving health infrastructures ex-ante rather than ex-post outbreaks remains a clear message for policy makers. Authors highlight that particularly in LMICs the funding of post-intervention research and the inclusion of an evaluation period in the design of the intervention are essential.

### Interventions for vector-borne diseases focused on housing and hygiene in urban settings: a scoping review [24]

The urban demographics explosion is a global, rapid and unavoidable phenomenon. It is therefore necessary to define and implement strategies of adaptation to cope with VBDs in urban settings. Such adaptation strategies will heavily rely on housing quality and urban public services (sanitation, rainwater management, access to drinking water, waste management) to limit vector abundance and reduce host-vector contact for VBDs prevention [25]. This evidence prompted the scoping review by Degroote et al. focusing on housing and hygiene interventions, including sanitation and waste management, to prevent VBDs in urban settings. Most of the 44 studies included in the review focused on *Aedes* mosquitoes and dengue transmission.

The authors noticed that multiple-component interventions have the potential to attain the widest and most sustainable public health impact. Control of mosquito breeding sites is the most widely implemented strategy and interventions such as house screening reveal promising, including for dengue control [26]. However, evaluation of interventions appears highly heterogeneous. Different types of indicators were used and in most cases, no epidemiological outcomes were reported and no comprehensive economic evaluation was performed.

Many studies reported on a positive effect of the interventions on mosquito populations – and especially a drop in larval populations. However, reduction of vector population is important but is far from being enough and there is currently no compelling evidence that a decrease in larval indices has an impact on the prevalence of VBDs such as dengue [27]. The authors therefore emphasize the importance of systematically assessing epidemiological indicators as the main objective of vector control remains the reduction of diseases burden.

Community and social mobilization are particularly promising for risk management at the household level. Nonetheless, social change is a complex process and several challenges have to be overcome to implement sustainable and large-scale actions [28]. Sustainability is stressed as a major issue and requires intersectoral partnership, advocacy at different levels, capacity building, human and financial resources. A robust monitoring and evaluation strategy based on quantitative and qualitative data is required to assess the progress achieved from a long-term perspective.

Waste management and sanitation, integration of ecological and sustainable vector control strategies, and implementation research were identified as research priorities. Finally, the use of standardized tools for conducting and reporting interventions is strongly encouraged as a way forward to increase comparability of the studies, ensure transferability of successful interventions and foster uptake of research results.

## Conclusions

The set of scoping reviews presented in this special issue presents a critical analysis of the state-of-the-art of research on urban health interventions for the prevention and control of vector-borne and other infectious diseases of poverty. The authors of the six reviews highlighted severe gaps in knowledge and identified organizational and theoretical limitations that need to be most urgently tackled to improve cities preparedness. Altogether, they call for more implementation research on VBDs in urban settings, grounded into carefully-thought, transferable designs and conducted according to shared standards. All scoping reviews consider that most vulnerable populations should be targeted as a matter of priority, especially if budgetary resources are limited, and any intervention should be systematically assessed on a regular basis.

Overall, scoping reviews recommendations for research and public health policy and practice for VBDs surveillance and control pertain to urban settings globally, whether cities are located in the South or in the North, in high-income or low-income countries. They address both the scientific community as well as policy makers and call for more political commitment and social mobilization through adequate citizen engagement strategies. Sharing experience and data and pooling resources hence appear the only way forward for the building of an optimal response to the pressing threat of VBDs on urban health.

## Additional file

Additional file 1: Multilingual abstracts in the six official languages of the United Nations. (PDF 674 kb)

## Abbreviations

IVM: Integrated Vector Management
LMICs: Low-and middle-income countries
TDR: The Special Programme for Research and Training in Tropical Diseases
VBDs: Vector-borne diseases
VERDAS: VEctor boRne DiseAses Scoping reviews
WHO: World Health Organization

## Footnotes

2 TDR, the Special Programme for Research and Training in Tropical Diseases, is a global programme of scientific collaboration that helps facilitate, support and influence efforts to combat diseases of poverty. It is hosted at the World Health Organization (WHO), and is sponsored by the United Nations Children’s Fund (UNICEF), the United Nations Development Programme (UNDP), the World Bank and WHO.

1 For further details please see paper in this special issue entitled “Establishing research priorities in prevention and control of vector-borne diseases in urban areas: a collaborative process” for more specific details on the methodology followed for prioritization purposes in the concept mapping exercise.
