# Supplementary material for: Supporting and strengthening research on urban health interventions for the prevention and control of vector-borne and other infectious diseases of poverty: scoping reviews and research gap analysis
Source: Infect Dis Poverty. 2018 Sep 3;7:94. doi: 10.1186/s40249-018-0462-z (PMC6120070; doi:10.1186/s40249-018-0462-z)
Supplement: Supplementary file 1 — Multilingual abstracts in the six official languages of the United Nations. (PDF 674 kb) [file 40249_2018_462_MOESM1_ESM.pdf]

Translation of the abstract into the six official working languages of the United Nations

دعم وتعزيز البحوث حول التدخلات الصحية (في المناطق) الحضرية للوقاية من الأمراض المنقولة بالنواقل وأمراض الفقر المعدية الأخرى والسيطرة عليها: مراجعات استطلاعية وتحليل للفجوة البحثية

مريم عثماني ديل باريو، فريديريك سيمارد، أندريا كابرارا

ملخص

الخلفية: يعيش أكثر من نصف سكان العالم حاليا في مستوطنات حضرية تنمو في الحجم والعدد. وبحلول عام 2050، سيعيش حوالي 70% من سكان العالم في تجمعات حضرية وبشكل خاص في البلدان المنخفضة والمتوسطة الدخل. إن التنقل والفقر واللامساواة بمستوياتها المختلفة وكذلك تقلب المناخ وتغيره لهي بعض من العوامل الاجتماعية والبيئية التي تؤثر على تعرض السكان في المناطق الحضرية للأمراض المنقولة بالنواقل التي تشكل تهديدات صحية عامة بارزة. هناك حاجة إلى تدخلات دقيقة ومتسقة ومبنية على الأدلة (للوقاية من الأمراض المنقولة بالنواقل وأمراض الفقر المعدية الأخرى والسيطرة عليها في المناطق الحضرية) من أجل تنفيذ سياسات عامة مبتكرة وفعالة من حيث التكلفة وتعزيز خدمات الصحة الشاملة والمتكافئة في المناطق الحضرية.

النص الرئيسي: على الرغم من وجود وعي متنامٍ بوبائيات الأمراض المنقولة بالنواقل على مستوى المناطق الحضرية، لا تزال هناك ندرة في البحوث والإجراءات التي تتبع في هذا المجال، مما يعوق القرارات والممارسات والاستراتيجيات (المتعلقة بسياسات الصحة العامة القائمة على الأدلة) التي تهدف لمشاركة نشطة للمجتمع المحلي. تصف هذه الورقة (البحثية) التعاون والشراكة بين برنامج منظمة الصحة العالمية الخاص بالبحوث والتدريب في مجال أمراض المناطق المدارية (TDR) والاتحاد/الائتلاف البحثي لمراقبة الأمراض المنقولة بالنواقل (VEctor-boRne DiseAses Scoping (VERDAS))، حيث تتضافر جهودهما في الاستجابة لسد الفجوة في المعرفة والأدلة من خلال دعم تطوير سلسلة المراجعات الاستطلاعية التي تسلط الضوء على الفجوات البحثية ذات الأولوية والآثار المترتبة على السياسات للتصدي للأمراض المنقولة والأمراض المعدية الأخرى على مستوى المناطق الحضرية.

الاستنتاجات: تقدم مجموعة المراجعات الاستطلاعية المقترحة في هذا العدد الخاص تحليلا نقديا لأحدث الأبحاث حول التدخلات الصحية في المناطق الحضرية من أجل الوقاية من الأمراض المنقولة بالنواقل وأمراض الفقر المعدية الأخرى. سلط مؤلفو المراجعات الستة الضوء على الفجوات الحادة في المعرفة وحدّدوا القيود التنظيمية والنظرية التي تحتاج إلى معالجة عاجلة لتحسين استجابة المدن واستجابة السيطرة على النواقل. إن الحاجة الأكثر إلحاحا في الوقت الحاضر هي ضمان إجراء المزيد من أبحاث التنفيذ حول الأمراض المنقولة بالنواقل في المناطق الحضرية، ومعالجة الآثار المترتبة على السياسات والممارسات، والدعوة إلى المزيد من الالتزام السياسي والتعبئة الاجتماعية من خلال استراتيجيات مشاركة كافية من جانب المواطنين.

Translated from English version into Arabic by Ammar Alhosain, proofread by Alaa Mahmoud, through

TRANSLATORS WITHOUT BORDERS

支持和加强城市卫生干预措施的研究以预防控制媒传疾病和其他贫困传染病: 勘域综述和研究差距分析

Mariam Otmani del Barrio, Frédéric Simard, Andrea Caprara

摘要

引言：目前，世界上有一半以上的人口居住在城市居民区，这些居民区的规模和数量都在增长。到 2050 年，全球约 70％的人口将居住在城市群中，主要集中在中低收入国家。流动性、贫困、不同层次的不平等以及气候多样性和变化性都是影响城市环境中人口暴露于媒传疾病的社会和环境因素，这些疾病构成了显着的公共卫生威胁。需要采取准确一致、循证的干预措施，预防和控制城市环境中的媒传疾病及其他贫困传染病，实施具创新性和成本效益的公共政策，使得城市卫生服务更加包容、更加公平。

主要内容：虽然人们越来越意识到城市地区媒传疾病流行病学面临问题，但在这一领域仍缺乏研究和行动，阻碍了循证公共卫生政策决策、实践以及战略的社区活动参与性。本文介绍了 WHO/TDR 和 VERDAS 研究联盟的合作与伙伴关系，他们通过撰写一系列勘域综述，共同努力填补知识和证据等方面的空白。这些综述突出了优先研究差距和政策影响，以应对城市中的媒传疾病和其他传染病。

结论：本专辑的一系列勘域综述对预防和控制媒传疾病和其他贫困传染病的城市卫生干预研究的最新技术进行了批判性分析。6 篇综述的作者强调了认知方面存在的明显差距，并确定了需要紧急应对的组织方面和理论方面的限制，以改善城市的应急准备工作和病媒控制反应。目前更为迫切的需要是确保对城市环境中的媒传疾病进行更多的研究，解决政策和实践问题，并通过适当的公民参与呼吁更多的政治承诺和社会动员。

Translated from English version into Chinese by Xue-Jiao Ma, edited by Pin Yang

CHINESE CENTER FOR DISEASE CONTROL AND PREVENTION
NATIONAL INSTITUTE OF PARASITIC DISEASES

Soutenir et renforcer la recherche sur les interventions de santé en milieu urbain pour la prévention et le contrôle des maladies à transmission vectorielle et autres maladies infectieuses de la pauvreté: examens de la portée et analyse des lacunes de la recherche.

Mariam Otmani del Barrio, Frédéric Simard, Andrea Caprara

Résumé
Contexte: Plus de la moitié de la population mondiale vit actuellement dans des zones urbaines, dont la taille et le nombre sont en augmentation. En 2050, environ 70 % de la population mondiale vivra dans des agglomérations, principalement dans les pays à revenu faible et intermédiaire. La mobilité, la pauvreté, les différentes couches d'inégalités ainsi que la variabilité et le changement climatiques sont quelques-uns des facteurs sociaux et environnementaux qui influencent l'exposition des populations urbaines aux maladies à transmission vectorielle, qui constituent d'éminentes menaces pour la santé publique. Des interventions précises, cohérentes et fondées sur des preuves pour la prévention et le contrôle des maladies vectorielles et autres maladies infectieuses dues à la pauvreté en milieu urbain sont nécessaires pour mettre en œuvre des politiques publiques innovantes et économiques et promouvoir des services de santé urbains inclusifs et équitables.
Discussion: Bien qu'il y ait une prise de conscience croissante de l'épidémiologie des maladies à transmission vectorielle en milieu urbain, les recherches et les actions entreprises dans ce domaine sont encore rares, ce qui empêche la prise de décisions politiques en matière de santé publique fondée sur les données probantes et les pratiques et stratégies d'engagement actif de la population. Cet article décrit la collaboration et le partenariat du Programme spécial de recherche et de formation sur les maladies tropicales (TDR) de l'OMS et du Consortium de recherche VERDAS, qui ont uni leurs efforts pour combler cette lacune dans les connaissances et les preuves en soutenant la réalisation d'une série de revues qui ont mis en évidence les lacunes des recherches prioritaires et les implications politiques dans la lutte contre les maladies à transmission vectorielle et autres maladies infectieuses en milieu urbain.
Conclusions: L'ensemble des revues de cadrage proposées dans ce numéro spécial présente un état des lieux critique de la recherche sur les interventions de santé en milieu urbain visant la prévention et le contrôle des maladies à transmission vectorielle et autres maladies infectieuses de la pauvreté. Les auteurs des six revues ont mis en évidence de graves lacunes dans les connaissances et identifié des limites organisationnelles et théoriques qui doivent être résolues d'urgence afin d'améliorer la préparation des villes et la lutte antivectorielle. Le besoin le plus pressant est de faire en sorte que davantage de recherches sur les maladies à transmission vectorielle en milieu urbain soient menées, en abordant les implications politiques et pratiques et en appelant à davantage d'engagement politique et de mobilisation sociale par des stratégies adéquates de mobilisation des citoyens.

Translated from English version into French by Callixte Nizigama, proofread by Suzanne Assenat, through

TRANSLATORS
WITHOUT BORDERS

Вспомогательное исследование по усилению оперативных здравоохранительных мер в городах с целью профилактики и борьбы с заболеваниями, передаваемых переносчиками, и другими инфекционными заболеваниями, сопутствующими бедности: обзорные статьи и анализ пробелов в исследованиях.

Мариам Отмани дель Баррио, Фредерик Симард, Андреа Капрара

Выдержка

Общая информация: В настоящее время более половины населения мира проживает в городских зонах, которые разрастаются, и количество которых увеличивается. К 2050 году примерно 70% мирового населения будет проживать в городских конгломератах, в основном в странах с низким и средним уровнем дохода населения. Мобильность, бедность, социальное неравенство среди населения, а также переменчивость климата и его изменение, — это часть тех социальных и экологических факторов, которые влияют на подверженность городских жителей заболеваниям, передаваемым переносчиками, представляющим собой большую угрозу для общественного здравоохранения. Для осуществления инновационной и экономически эффективной государственной политики требуются точные и последовательные оперативные меры по профилактике и борьбе с заболеваниями, передаваемых переносчиками, и другими инфекционными заболеваниями, сопутствующими бедности, эффективность которых доказана. Также требуется поощрение работы городских органов здравоохранения на основе инклюзии и равноправия.
Основной текст: Несмотря на растущую осведомленность об эпидемиологии заболеваний, передаваемых переносчиками, в городах, по-прежнему существует нехватка исследований и мер, предпринимаемых в этой области, что препятствуетпринятию политических решений в области общественного здравоохранения на доказательной основе, а также внедрению стратегий и практики по активному вовлечению населения. В данном документе описывается сотрудничество и партнерство Специальной программы ВОЗ по исследованиям и обучению кадров в области тропических болезней (Training in Tropical Diseases, TDR) и Исследовательского консорциума VERDAS, которые объединили свои усилия, чтобы заполнить пробелы в знаниях и фактических данных путём создания обзорных статей, в которых подчеркиваются пробелы в исследованиях, для которых требуется проведение исследований в первую очередь, а также содержатся практические выводы для разработки политики в области здравоохранения по борьбе с заболеваниями, передаваемыми переносчиками, и другими инфекционными заболеваниями в городах.
Заключение: Подборка обзорных статей, предложенная в этом специальном выпуске, представляет собой критический анализ новейших исследований о мерах, предпринимаемых в городах для профилактики и борьбы с заболеваниями, передаваемыми переносчиками, и другими инфекционными заболеваниями, сопутствующими бедности. Авторы этих шести обзорных статей сконцентрировались на серьезных пробелах в знаниях и определении организационных и теоретических ограничений, которые необходимо срочно преодолеть для того, чтобы города были лучше подготовлены к борьбе с переносчиками инфекций. Самой насущной потребностью в настоящее время является обеспечение проведения большего количества исследований в области заболеваний, передаваемых переносчиками в городских условиях, для разработки политики и практических решений в сфере здравоохранения, а также призыв к большей политической ответственности и социальной мобилизации путем внедрения соответствующих стратегий по привлечению населения.

Translated from English version into Russian by Roman Maksimov, proofread by Oksana Weiss, through

TRANSLATORS WITHOUT BORDERS

Apoyo y fortalecimiento de la investigación sobre intervenciones de salud urbana para la prevención y el control de las enfermedades transmitidas por vectores y otras enfermedades infecciosas de la pobreza: revisiones de alcance y análisis de brechas en la investigación.

Mariam Otmani del Barrio, Frédéric Simard, Andrea Caprara

Resumen

Antecedentes: Actualmente, más de la mitad de la población mundial vive en asentamientos urbanos que crecen tanto en tamaño como en número. Para 2050, aproximadamente el 70 % de la población mundial vivirá en conglomerados urbanos, principalmente en países con ingresos bajos y medianos. La movilidad, la pobreza y las diferentes capas de desigualdad, así como la variabilidad y el cambio climáticos, son algunos de los factores sociales y ambientales que influyen en la exposición de las poblaciones humanas en entornos urbanos a las enfermedades transmitidas por vectores, que presentan reconocidas amenazas para la salud pública. Las intervenciones exactas, consistentes y con base empírica para la prevención y el control de enfermedades transmitidas por vectores y otras

enfermedades infecciosas de la pobreza en entornos urbanos son necesarias para implementar políticas públicas innovadoras y rentables, así como para fomentar los servicios de salud urbana incluyentes e igualitarios.
Parte principal: Si bien existe una mayor conciencia de la epidemiología de las enfermedades transmitidas por vectores a nivel urbano, todavía hay una escasez de investigaciones y toma de medidas en esta área, lo que obstaculiza las decisiones sobre políticas de salud pública con base empírica, y la práctica y las estrategias para la participación activa de la comunidad. Este trabajo describe la colaboración y asociación entre el Programa Especial de Investigaciones y Enseñanzas sobre Enfermedades Tropicales (TDR) de la OMS y el Consorcio de Investigación VERDAS, que aunaron esfuerzos en respuesta a la necesidad de llenar este vacío de conocimiento y evidencia apoyando el desarrollo de una serie de revisiones del alcance que ponen de relieve las brechas de investigación prioritarias y las implicaciones de políticas para dar respuesta a las enfermedades transmitidas por vectores y otras enfermedades infecciosas a nivel urbano.
Conclusiones: El conjunto de revisiones de alcance propuestas en este número especial presenta un análisis crítico de la investigación de avanzada sobre las intervenciones de salud urbana para la prevención y el control de enfermedades transmitidas por vectores y otras enfermedades infecciosas de la pobreza. Los autores de las seis revisiones destacaron brechas graves de conocimiento e identificaron limitaciones organizativas y teóricas que deben abordarse con urgencia para mejorar la preparación de las ciudades y la respuesta de control de los vectores. La necesidad más acuciante en la actualidad es garantizar que se realicen más investigaciones de implementación sobre las enfermedades transmitidas por vectores en entornos urbanos, que aborden las implicaciones de políticas y prácticas, y convoquen a que haya más compromiso político y movilización social a través de estrategias adecuadas de participación ciudadana.

Translated from English version into Spanish by Susana Rosselli, proofread by Macarena Belén Pierrot, through

TRANSLATORS
WITHOUT BORDERS
